# Commentary: Feeling the Conflict: The Crucial Role of Conflict Experience in Adaptation

**DOI:** 10.3389/fpsyg.2017.01405

**Published:** 2017-09-11

**Authors:** Anna Foerster, Roland Pfister, Heiko Reuss, Wilfried Kunde

**Affiliations:** Department of Psychology III, University of Würzburg Würzburg, Germany

**Keywords:** conflict adaptation, conflict experience, conflict strength, cognitive conflict, cognitive control

Conflict adaptation in masked priming has recently been proposed to rely not on successful conflict resolution but rather on conflict experience (Desender et al., 2014). We re-assessed this proposal in a direct replication and also tested a potential confound due to conflict strength. The data supported this alternative view, but also failed to replicate basic conflict adaptation effects of the original study despite considerable power.

## Introduction

Unconscious stimuli can activate motor responses, which causes cognitive conflict if this activation does not match what the agent intends to do. Fortunately, the human cognitive system adapts to conflicts, an ability that manifests in congruency sequence effects (Botvinick et al., 2001). These effects are typically taken to indicate that successfully overcoming cognitive conflict reduces conflict in the following trial.

Desender et al. (2014) argued against this view by proposing that subjective conflict experience—instead of actual conflict—is the driving force of conflict adaptation. This claim was based on a study in which participants rated conflict experience after responding to a target arrow that followed a barely visible congruent or incongruent prime arrow. Sequence effects were evident after accurate conflict ratings, but absent or reversed after incorrect conflict ratings. These data demonstrate that conflict can become consciously available. However, the observation of common changes in subjective experience and conflict adaptation is correlational in nature and leaves room for explanations in terms of additional variables that determine *both*, conflict experience and conflict adaptation. A candidate variable is the actual *conflict strength* (i.e., strength of activation of competing responses) that temporally precedes both other measures (Forster et al., 2011; Wendt et al., 2014; Abrahamse and Braem, 2015; Hommel, 2017).

The above-chance detection of congruent and incongruent trials via conflict ratings in the original study already supports the notion of a direct impact of conflict strength on conflict experience. However, it cannot account for variations of conflict adaptation by other factors than congruency. Analyzing adaptation effects as a function of conflict experience might have tapped also into unsystematic sources of conflict (Abrahamse and Braem, 2015), leading to increased conflict strength in trials with higher ratings of conflict. Performance speed (response times, RTs) should serve as an approximation for such unsystematic influences of conflicting responses (plus systematic influences of congruency) as responses to incongruent compared to congruent stimuli are slower and lead to higher ratings of conflict. Accordingly, we replicated the original study and scrutinized the correlational relation of performance speed and conflict experience.

## Methods

The study closely matched the original procedure. A masked prime arrow preceded a target arrow on each trial and participants responded to the direction of the target arrow (see the Supplementary Material for details). The only differences to the original methodology were that we presented error feedback in the main task and asked for conflict ratings only after correct responses rather than deleting those trials afterward. Of the 89 participants (power ≥ 80% for all relevant effects, see the Supplementary Material), three participants had to be excluded following the criteria of the original study. The study was conducted in accordance with the guidelines of the ethics committee at the Institute of Psychology at the University of Würzburg, as well as with the guidelines of the Deutsche Gesellschaft für Psychologie (German Psychological Society). All participants gave written informed consent prior to participation.

## Results

### Conflict rating

**Figure 1:**
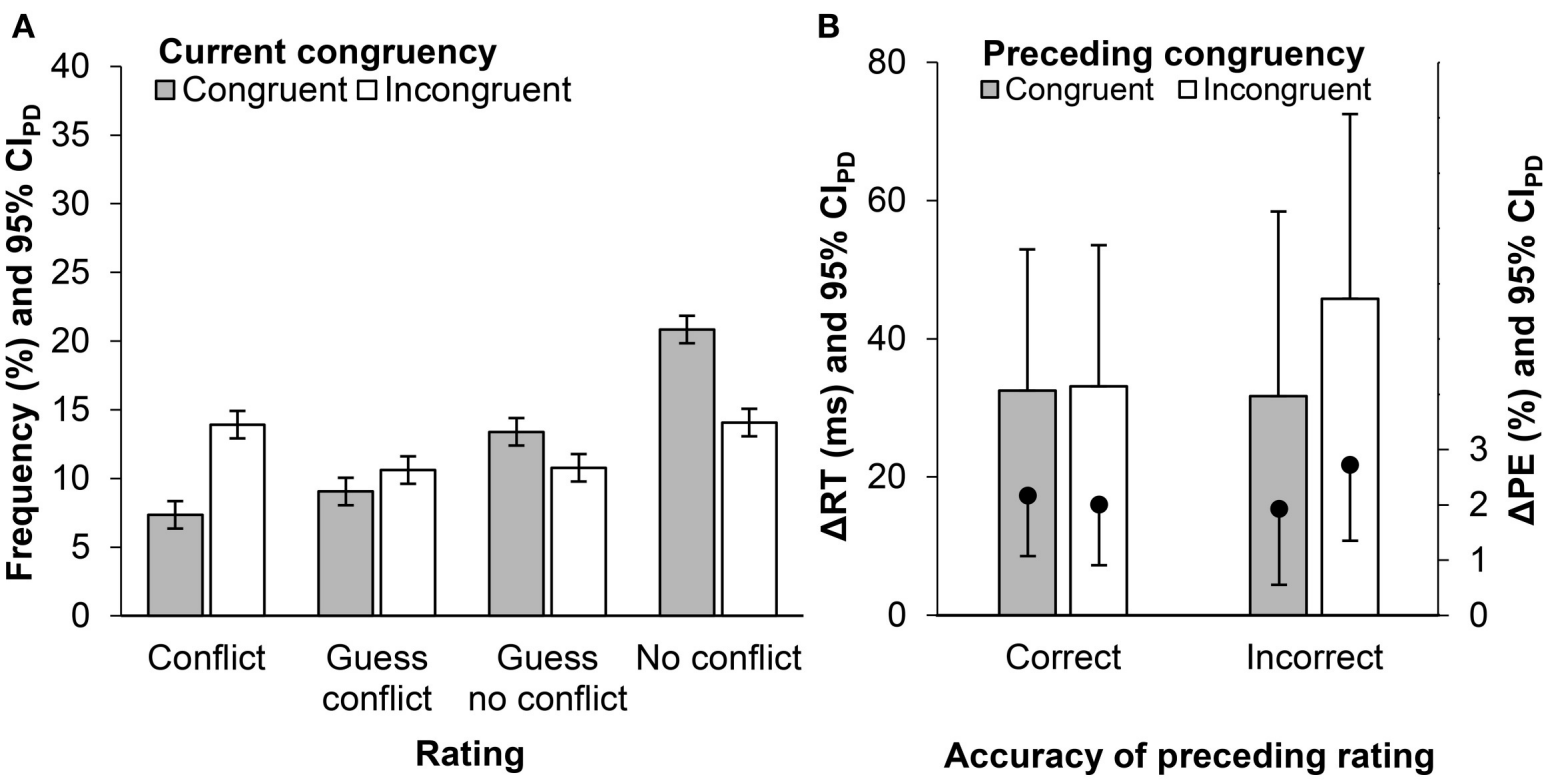
**(A)** Conflict rating frequencies for congruent and incongruent trials. **(B)** Effects of current congruency (Δ = currently incongruent – currently congruent) in reaction times (ΔRT; bars) and error percentages (ΔPEs; dots), as a function of preceding congruency and rating accuracy (see Figure S1 for raw RTs and PEs). Error bars represent the 95% confidence interval of paired differences (CI_PD_; Pfister and Janczyk, 2013), computed separately for each rating condition.

Ratings were evenly distributed across the four categories “There was conflict,” “I guess there was conflict,” “I guess there was no conflict,” and “There was no conflict” (Figure 1A). Thus, we dichotomized all four ratings in “conflict” and “no conflict.” Twenty-three participants did not provide sufficient observations and were excluded following the original methodology. The remaining participants detected conflict better than chance (mean conflict-d' = 0.49, *SD* = 0.48), *t*(62) = 8.09, *p* < 0.001, *d*_*z*_ = 1.02.

### Performance speed and conflict rating

Unstandardized participant-wise regression coefficients predicting conflict ratings by RTs (in seconds) were tested against zero (mean slope = 0.52, *SD* = 0.60). Positive values indicate a tendency toward conflict for slower RTs and this tendency was significant, *t*(62) = 6.85, *p* < 0.001, *d*_*z*_ = 0.86. We repeated this analysis for incongruent trials (objective presence of conflict) which resulted in very similar results (mean slope = 0.51, *SD* = 0.63), *t*(62) = 6.51, *p* < 0.001, *d*_*z*_ = 0.82^1^.

### Congruency sequence effect and conflict rating

RTs and error percentages (PEs) of the target response were analyzed in 2 × 2 × 2 analyses of variance (ANOVAs) with the factors current congruency, preceding congruency and accuracy of the preceding rating (Figure 1B). Congruent responses were faster, *F*(1, 62) = 74.89, *p* < 0.001, $\eta_{p}^{2}=$ 0.55, and more accurate, *F*(1, 62) = 34.63, *p* < 0.001, $\eta_{p}^{2}$ = 0.36, than incongruent responses. None of the remaining effects were significant, *F*s ≤ 2.47, *p*s ≥ 0.121.

## Discussion

Surprisingly, our data did not yield any signs of conflict adaptation even after correct conflict ratings (see Supplementary Material for Bayesian follow-up analyses). Given that our method was a direct replication of Desender et al. (2014), this finding is puzzling and raises the question of whether our results might reflect a statistical Type II error or a possible Type I error in the original study. The former error seems unlikely given the clear absence of adaptation effects despite relatively high statistical power (higher than in the original study; see the Supplementary Material). Possibly, the present and the original study design hindered potential sequential effects, because two target responses are separated by a relatively long time and by several unrelated responses (the conflict rating and an additional response to start the next trial). A re-assessment of additional data sets that allow for similar analyses (e.g., Desender et al., 2016) might shed further light on this question.

The above-chance detection of congruent and incongruent trials by ratings in both studies shows a direct impact of conflict strength on conflict experience. The correlation of performance speed and conflict ratings in the current study suggests that unsystematic conflict sources could contribute to conflict strength and, thus, to conflict experience (Abrahamse and Braem, 2015).

The evidence of conflict adaptation in masked priming designs is ambiguous as adaptation is sometimes found whereas it is absent at other times (for a review, see Kunde et al., 2012; Ansorge et al., 2014). The current data reveals that the consideration of conflict experience does not render conflict adaptation more reliable. Third variables like affective experience of conflict (Fröber et al., 2017) or conflict strength could be potential sources of conflict adaptation. Future studies should manipulate conflict strength systematically (e.g., Eimer and Schlaghecken, 1998) to observe causal relations of conflict strength, experience and adaptation of subliminal information.

## Author contributions

RP proposed the research question and all authors discussed and agreed on the methodological approach. AF and RP acquired and analyzed data and drafted the manuscript. All authors revised the work, approved the final version for publication, and agree to be accountable for all aspects of the work.

### Conflict of interest statement

The authors declare that the research was conducted in the absence of any commercial or financial relationships that could be construed as a potential conflict of interest.

## Footnotes

^1^We thank one of the reviewers for suggesting this analysis.
