# Supplementary material for: Commentary: Feeling the Conflict: The Crucial Role of Conflict Experience in Adaptation
Source: Front Psychol. 2017 Sep 11;8:1405. doi: 10.3389/fpsyg.2017.01405 (PMC5601984; doi:10.3389/fpsyg.2017.01405)
Supplement: Supplementary file 1 [file DataSheet1.pdf]

Supplementary Material

Commentary: Feeling the Conflict: The Crucial Role of Conflict Experience in Adaptation

Anna Foerster*, Roland Pfister*, Heiko Reuss and Wilfried Kunde

* These authors have contributed equally to this work. Correspondence: anna.foerster@uni-wuerzburg.de or roland.pfister@psychologie.uni-wuerzburg.de

1. Supplementary Methods

1.1. Participants

A range of different effects from the original study can be considered for estimating an optimal sample size for a direct replication of Desender et al. (2014). These effects comprise the three-way interaction of current congruency, preceding congruency, and accuracy of the preceding rating for reaction times (RTs; $d_z = 0.30$) and error percentages (PEs; $d_z = 0.47$), and the robust congruency sequence effects after correct conflict ratings (RTs: $d_z = 0.45$; PEs: $d_z = 0.45$). Effect sizes were computed from the corresponding F-statistics and we chose to base our sample size on the lowest of these values ($d_z = 0.30$). A sample size of 89 participants ensures a power of 80% to detect such an effect in a two-tailed test (with $\alpha = 5\%$; calculated with the *power.t.test* function in *R3.1.0*). This sample size also allows for sufficient power (> 90%) for the stronger effects even after a high expected dropout rate of 40% following the criteria of the original study. Three participants had to be excluded as their median RT exceeded the sample mean by two SDs. The remaining 86 participants (19 male) met the accuracy inclusion criterion (less than 25% errors; see below for exclusions based on trial numbers).

1.2. Apparatus and Stimuli

Participants sat in front of a 17’’ CRT monitor and responded on a standard QWERTZ keyboard. We set the vertical refresh rate to 85 Hz and the background was white. Prime and target arrows as well as the masks were identical to the original stimulus set (all materials are openly available on the Open Science Framework, osf.io/ghh7e). Participants responded to targets with a left (key D) or right (key K) key press with their left and right index finger. Conflict ratings were given by pressing one of the number keys from 1 to 4. Participants started each trial via a key press on the space bar.

1.3. Procedure

Participants were instructed to press the left key for a left-pointing target arrow and the right key for a right-pointing target arrow. The instruction equally emphasized fast and accurate responses. In addition, participants learned about the very short presentation of a barely visible arrow before the target arrow and that these two arrows might be conflicting. Participants were told that this could lead to an impression of slowed responding, to false responding or to a feeling that something is strange. Participants had to indicate after each correct trial whether they thought there had been a conflict. Participants had to select one of the following responses (in German): “(1) I think there was a conflict”, “(2) I don’t know (but I guess there was a conflict)”, “(3) I don’t know (but I guess there was no conflict)” and “(4) I think there was no conflict”. In the following,

these options are shorty referred to as “conflict”, “guess conflict”, “guess no conflict” and “no conflict”.

Participants pressed the space bar to commence with a trial. Then, a fixation cross was presented in the center of the screen for 1000 ms. A prime arrow appeared in the center of the screen for 23 ms. The two masks were presented for 23 ms each. After the presentation of the two masks, the screen remained blank for another 23 ms. The target appeared in the center of the screen for 160 ms and participants were to respond within 3000 ms. Early responses, response omissions or commissions (i.e., right key press to a left-pointing arrow or left key press to a right-pointing arrow) were acknowledged in an error-specific feedback for 1000 ms. In contrast to Desender et al. (2014), error feedback appeared in each trial (except in the last signal detection block, see below for more information). The reason for this change was to reduce data exclusion rate. This strategy was successful considering that no participant had to be excluded because of a high error rate when applying the same criterion as in the original study. In case of a correct response, the conflict rating display followed participants’ target response.

Participants started with a practice block of eight trials without conflict ratings, followed by a practice block of 40 trials with conflict ratings. Afterward, participants went through eight blocks of 60 trials each. In a last signal detection block of 100 trials, participants responded to the prime arrow without giving a conflict rating. This last block was included to assess prime visibility. Participants could take self-paced breaks between blocks.

2. Data Treatment, Analyses, and Supplementary Results

The complete data set and the commented analysis scripts are available on the Open Science Framework (osf.io/ghh7e).

2.1. Conflict Rating

Only trials with correct target responses were considered for the analysis of conflict detection, (3.9% erroneous trials excluded). Incongruent trials (signals) which participants correctly rated as conflicting were defined as hits. Congruent trials (noise) which participants falsely rated as conflicting were defined as false alarms. The hit rate was computed for each participant by dividing the number of hits by the number of signals. The number of false alarms was divided by the number of noise trials to get false alarm rates.[1] Hit or false alarm rates of 0 were corrected to .05 and rates of 1 were corrected to .95 for nine participants. The discriminability index conflict-d’ (i.e., detection accuracy of congruent and incongruent trials) was computed as z(hit rate)-z(false alarm rate). The response bias index conflict-c (i.e., bias to indicate more frequently that there was a conflict than that there was no conflict or vice versa) was computed as -0.5*[z(hit rate)+z(false alarm rate)]. Conflict-d’ and conflict-c were both tested against 0 in one-sample *t*-tests.

All 86 participants were able to discriminate congruent and incongruent trials better than chance (conflict-d’ = 0.51, SD = 0.63), $t(85) = 7.51$, $p < 0.001$, $d_z = 0.81$, and they gave more not conflicting than conflicting judgements (conflict-c = 0.31, SD = 0.81), $t(85) = 3.54$, $p = 0.001$, $d_z = 0.38$. The final sample did not show any bias (mean conflict-c = 0.09, SD = 0.44), $t(62) = 1.62$, $p = 0.110$, $d_z = 0.20$, and their discrimination performance was comparable to that of the full sample (see the main text).

[1] Desender et al. (2014) divided the number of false alarms by the number of *signals*. Statistical analyses included less signals than noises because errors were more frequent in incongruent than in congruent trials. This leads to an overestimation of the false alarm rate and, in turn, to an underestimation of conflict sensitivity.

2.2. Performance Speed and Conflict Rating

The first trial of each block and trials that followed errors (3.8%) were excluded. A linear regression analysis with current RT as predictor and the resulting rating as criterion assessed whether the RT influenced conflict ratings following common procedures for regression coefficient analysis (Lorch & Meyers, 1990; Pfister, Schwarz, Carson, & Janczyk; 2013).

2.3. Congruency Sequence Effect and Conflict Rating

PEs were computed as the ratio of commission errors to commission errors plus accurate target responses. Figure S1 depicts the mean PEs and RTs of all experimental conditions. On average, it took participants 548 ms (SD = 570.24) to start a trial and 773 ms (SD = 709.52) to deliver a conflict rating. JZS-Bayes-factors (BFs; calculated with the *BayesFactor* package version *0.9.12-2* running in *R3.3.0*) indicate substantial evidence for absent three-way interactions in RTs (BF = 5.56) and PEs (BF = 4.49) and for no adaptation effects after correct ratings in RTs (BF = 7.23) and PEs (BF = 6.94).

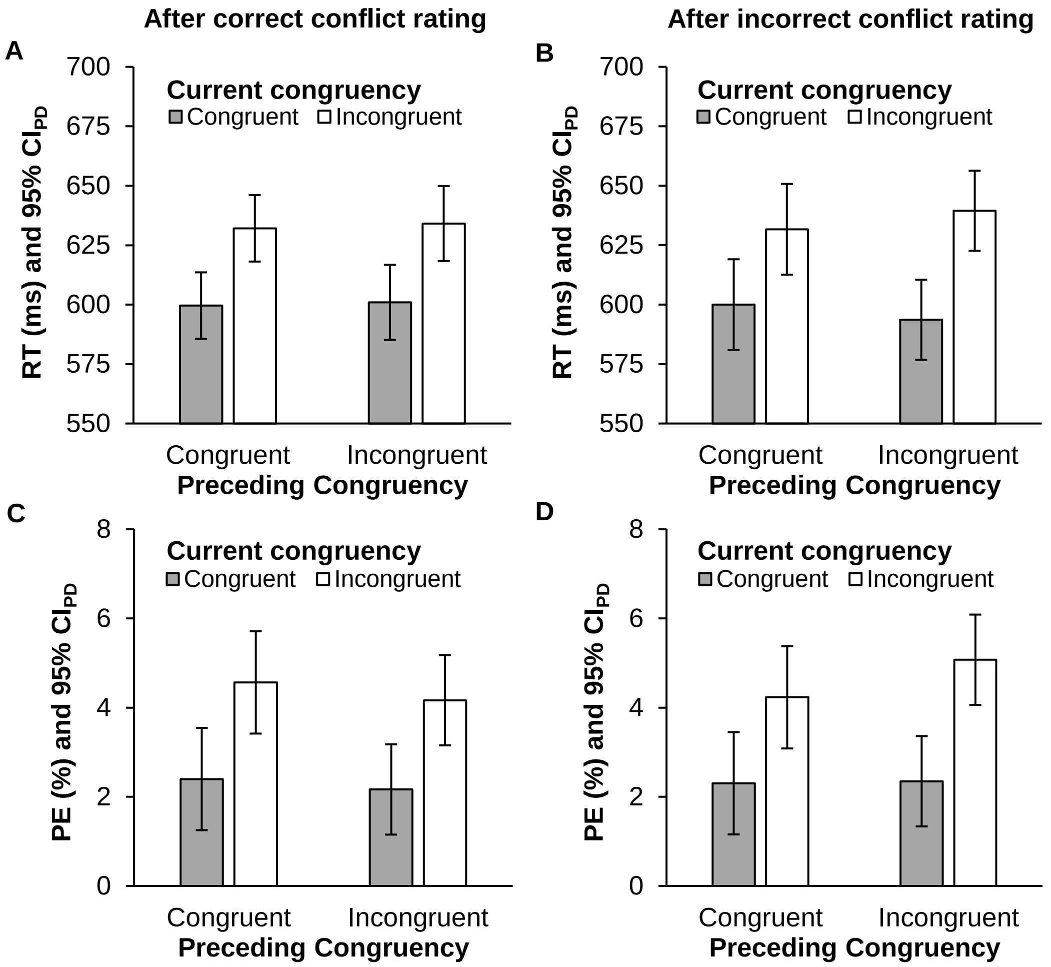

Figure S1 | Mean reaction times (RTs; upper panels) and error percentages (PEs; lower panels) of the target response, plotted as a function of current congruency, preceding congruency and preceding rating accuracy with correct conflict ratings in the left panels and incorrect conflict ratings in the right panels. Participants responded faster and more accurately in congruent than in incongruent trials. Error bars represent the 95% confidence interval of paired differences (CI_{PD}; Pfister & Janczyk, 2013), computed separately for each comparison of currently congruent and currently incongruent trials.

2.4. Prime Visibility

To assess whether participants were able to discriminate primes, we analyzed prime responses in the signal detection block (response omissions and early responses were excluded: 1.2%). Left-pointing primes which participants correctly identified as left-pointing were defined as hits. Right-pointing primes which participants falsely identified as left-pointing were defined as false alarms. The hit rate was computed for each participant by dividing the number of hits by the number of signals. The number of false alarms was divided by the number of noise trials to get false alarm rates. Corrections were not necessary. Visibility-d' (i.e., detection accuracy of left-pointing and right-pointing primes) was computed as z(hit rate)-z(false alarm rate). The response bias index visibility-c (i.e., bias to indicate more frequently that there was a left-pointing than a right-pointing prime or vice versa) was computed as -0.5*[z(hit rate)+z(false alarm rate)]. Visibility-d' and visibility-c were both tested against 0 in one-sample *t*-tests.

Participants were able to discriminate primes better than chance (mean visibility-d' = 0.13, SD = 0.20), $t(62) = 5.14$, $p < 0.001$, $d_z = 0.65$, and they identified prime arrows more often as right-pointing than left-pointing (mean visibility-c = 0.08, SD = 0.22), $t(62) = 2.90$, $p = 0.005$, $d_z = 0.37$. Figure S2 depicts the relation of visibility-d' and conflict-d', and the Pearson correlation of both variables was significant, $r = 0.32$, $t(62) = 2.67$, $p = 0.010$. The intercept of the corresponding linear regression analysis was also significant (with visibility-d' = 0.39), $t(62) = 5.64$, $p < 0.001$. Pearson correlations between visibility-d' and the adaptation effects after correct or incorrect ratings were not significant, $|rs| \leq 0.10$, $ps \geq 0.450$.

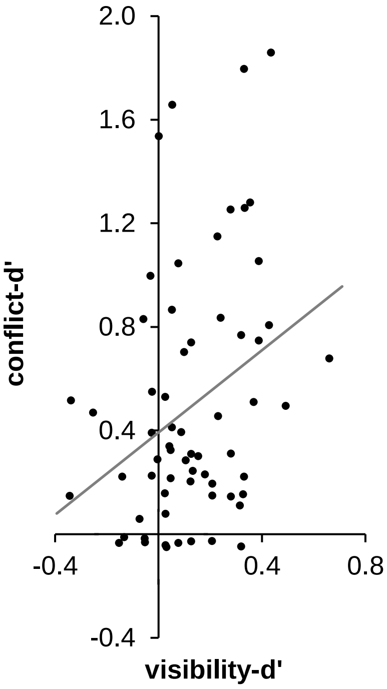

Figure S2 | Relation of visibility-d' (x-axis) and conflict-d' (y-axis) and its linear regression line (y = 0.79x + 0.39). There was a positive correlation but conflict-d' was also above chance level at zero prime visibility.

3. Supplementary References

Lorch, R. F., & Myers, J. L. (1990). Regression analyses of repeated measures data in cognitive research. Journal of Experimental Psychology: Learning, Memory, and *Cognition, 16*(1), 149-157. doi: 10.1037/0278-7393.16.1.149

Pfister, R., & Janczyk, M. (2013). Confidence intervals for two sample means: Calculation, interpretation, and a few simple rules. *Advances in Cognitive Psychology*, *9*(2), 74–80. doi:10.2478/v10053-008-0133-x

Pfister, R., Schwarz, K. A., Carson, R., & Janczyk, M. (2013). Easy methods for extracting individual regression slopes: Comparing SPSS, R, and Excel. *Tutorials in Quantitative Methods for Psychology*, *9*(2), 72-78.
